# *QuickStats*: Percentage* of Total Daily Kilocalories^†^ Consumed from Sugar-Sweetened Beverages^§^ Among Children and Adults, by Sex and Income Level^¶^ — National Health and Nutrition Examination Survey, United States, 2011–2014

**DOI:** 10.15585/mmwr.mm6606a8

**Published:** 2017-02-17

**Figure:**
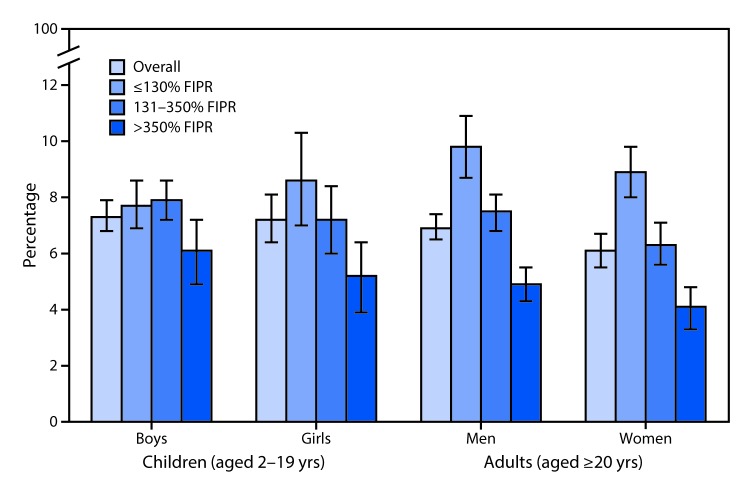
During 2011–2014, on average, 7.3% of boys’ and 7.2% of girls’ total daily calories were obtained from SSBs compared with 6.9% for men and 6.1% for women. For men, women, and girls, the percentage of total daily kilocalories from SSBs declined as income level increased. For boys, the percentage of total daily kilocalories was lower for those in the highest income group than in the other income groups. Compared with women, a larger proportion of men’s total daily kilocalorie intake came from SSBs.

**Source:** Rosinger A, Herrick K, Gahche J, Park S. Sugar-sweetened beverage consumption among US adults, 2011–2014. NCHS Data Brief no. 270; 2017. https://www.cdc.gov/nchs/products/databriefs/db270.htm.

Rosinger A, Herrick K, Gahche J, Park S. Sugar-Sweetened beverage consumption among US youth, 2011–2014. NCHS Data Brief no. 271; 2017. https://www.cdc.gov/nchs/products/databriefs/db271.htm.

